# Factors Associated with the Prevalence of Psychiatric Disorders Among Saudi Adults in the Eastern Region and Their Health Implications

**DOI:** 10.3390/healthcare12232419

**Published:** 2024-12-02

**Authors:** Kholoud B. Alabdulkareem, Ghedeir M. Alshammari, Ali Abdullah Alyousef, Mohammed A. Mohammed, Sndos Z. Fattiny, Ismail Zayed Alqahtani, Mohammed Abdo Yahya

**Affiliations:** 1 Department of Social Studies, College of Arts, King Saud University, Riyadh 11495, Saudi Arabia; kabdulkreem@ksu.edu.sa; 2 Department of Food Science and Nutrition, College of Food and Agricultural Sciences, King Saud University, Riyadh 11451, Saudi Arabia; muhammed-awad@hotmail.com (M.A.M.); 442204496@ksu.edu.sa (S.Z.F.); mabdo@ksu.edu.sa (M.A.Y.); 3 Department of Clinical Nutrition, Mental Health Hospital Al-Ahsa, Minister of Health, Hofuf 13791, Saudi Arabia; alaalyousef@moh.gov.sa (A.A.A.); ismaeel626@gmail.com (I.Z.A.)

**Keywords:** psychiatric, Al-Hassa, body composition, sociodemographic, anthropometric, hematological parameters

## Abstract

Background/Objectives: The present study examined the link between sociodemographic variables, anthropometric indices, and psychiatric disorders among patients in Saudi Arabia’s Eastern Region (Al-Hassa), as well as health outcomes, including basic hematological and biochemical markers. Methods: The patients included 89 females and 79 males with psychiatric disorders, with ages ranging from 19 to 59. Sociodemographic characteristics, anthropometric proxies, and fundamental hematological and biochemical markers were assessed. Results: The sociodemographic characteristics of the patients were poor and varied within and between sexes. This study observed that male psychiatric patients had greater anthropometric proxies, particularly those who were overweight or obese, than females. Most of the patients’ hematological and biochemical parameters were below the normal level, with some higher than normal. Moreover, anemia was identified in 40.51% of the male participants in the study, with a higher percentage among those diagnosed with depressive disorders (Dep-d, 57.14%) and schizophrenia spectrum and other psychotic disorders (SsP-d, 32.43%), and 49.44% of the female participants, with a higher percentage among those diagnosed with depressive disorders (52.50%) and other psychotic disorders (46.15%). Furthermore, to confirm the link between sociodemographic variables, anthropometric indices, and psychiatric disorders among patients, the Spearman correlation coefficient and simple regression analysis of such variables was carried out. The results revealed that the majority of sociodemographic characteristics were either favorably or adversely correlated with patients’ anthropometrics and type of depression in both sexes. Conclusion: Low sociodemographic characteristics and high anthropometric variables may be risk factors for people with psychotic disorders, which have been linked to negative health consequences.

## 1. Introduction

Globally, the number of older people suffering from major depressive disorder is on the rise. Major depression in the elderly is influenced by several clinical and demographic factors, including age and gender. The quality of life and mental health of the elderly are therefore crucial [1]. Moreover, Lackamp et al. [2] reported that major depressive disorder is one of the most common mental disorders worldwide and is prevalent across the lifespan, with prevalence estimates ranging from 1 to 5% in people aged 65 years or older. The older population is growing both in absolute numbers and as a percentage of the total world population, including those with bipolar disorder, major depressive disorder, schizoaffective disorder, or schizophrenia [3]. Although individuals with major depressive disorder, bipolar disorder, and schizophrenia die prematurely, some with these conditions live to an advanced age and may have significant physical health burdens and multiple morbidities, predisposing them to require nursing home admission [4].

According to the Saudi National Mental Health Survey (SNMHS), 34.2% of Saudis have been diagnosed with a mental disorder at some time in their lives [5]. According to Altwaijri et al. [6], the most frequent mental health illnesses in the Kingdom of Saudi Arabia (KSA) are anxiety, mood, disruptive behavior, eating, and drug use disorders. According to Noorwali et al. [7], there are various obstacles to prompt and effective care for mental health issues, including stigma, a lack of awareness, and a lack of quality care. Furthermore, those who recognized the need for therapy stated that they preferred to manage the matter on their own [8]. In the study by Alzahrani et al. [9], female gender, a younger age group, single/divorced marital status, lower educational level, lower socioeconomic status, non-Saudi residential status, student status, small family size, and elderly house member status are predictors of mental illness. A study of the student population indicated that a lack of physical exercise and insufficient sleep were the main determinants of poor mental health [10]. In addition, smoking, poor health, and a lack of resilience have been linked to mental health issues in Saudi Arabia [9].

The Eastern Province is the biggest province in Saudi Arabia in terms of land and one of the most important because it is the most industrialized part of the Kingdom and the world’s third-biggest oil-producing region [11]. The industrialization and fast urbanization of the Eastern Province have resulted in lifestyle changes among residents. Lifestyle changes such as unhealthy food consumption and decreased physical activity appear to be associated with the general body weight of students in the Eastern Province, with those reported to be overweight ranging from 11.7% to 20.5% and obese from 9.5% to 20.5% [12]. A study conducted by Woodman et al. [11] observed that the lifestyles in Saudi Arabia’s Eastern Province may have long-term negative health consequences, which is significant given the Kingdom’s rising rates of overweight, obesity, and obesity-related non-communicable illnesses among its adult and aging population. According to Alhusseini et al. [13], Saudi Arabia’s urbanization and economic growth have resulted in lifestyle changes such as increased sedentary behavior and a trend toward more energy-dense foods. These changes, which are frequently associated with urban living, have contributed to the obesity pandemic. Al Nasser et al. [14] found a strong relationship between obesity and raised BMI, as well as a higher prevalence of depression and anxiety among individuals. Around a third of the participants had signs of depression, with another one-third having symptoms of an anxiety disorder. An investigation found that the female gender was more likely to have a mental illness, which was significantly correlated with obesity, diabetes, and strokes [15]. This was because women are reported to worry more frequently and experience increasing levels of discomfort and fear. In women, biological differences, especially menstrual patterns, have an impact on mood and coping skills. Overall, men and women develop mental health issues differently, with a complicated interaction between hereditary and environmental factors [16].

The blood parameters, fundamental pathogenic mechanism, and patient history should all be considered when determining categorization and diagnosis [17]. When left untreated, anemia can cause fatigue and heart palpitations, as well as disturbances in cognitive function and depression that are accompanied by psychological disorders [18]. Akaishi et al. [19] found that reduced Hb levels and raised neutrophil-to-lymphocyte, monocyte-to-lymphocyte, and platelet-to-lymphocyte ratios had a significant effect on depression symptoms and sleep disruptions in the general population. These relationships remained significant even after accounting for possible confounders such as age, gender, BMI, and daily tobacco use. Males and females both showed associations, although males showed them more prominently. Research has suggested a link between chronic inflammation and psychiatric diseases such as mood disorders and schizophrenia [20]. Nevertheless, the exact association between circulating blood cell patterns and mental health issues such as depression and sleep disturbances in the general population is unknown. Low hemoglobin (Hb) levels have been shown to have a deleterious impact on a wide range of psychiatric diseases, including depression and cognitive functions [21,22]. Many factors influence Hb levels, including sex, age, and smoking status [23]. Vitamin B12 deficiency and folate deficiency anemias are conditions in which not enough healthy red blood cells are created to transport oxygen throughout the body, and their absence can cause several illnesses [24]. Because gathering comprehensive information from a large number of people is challenging, the precise association between low Hb levels and mental disorders in the general population is unknown [19]. Furthermore, the effect of various blood cell profiles, such as white blood cell count (WBC), WBC subpopulations, platelet count, and derived inflammatory hematological ratios on mental health is mainly unknown [19]. The present study examined the association between psychiatric disorders, anthropometric indices, and sociodemographic traits among patients in the Eastern Region (Al-Hassa) of Saudi Arabia. Additionally, the study examined health outcomes, including basic hematological and biochemical markers.

## 2. Material and Methods

### 2.1. Subjects and Sample Size

A cross-sectional study was conducted to collect data from psychiatric patients at a specific point in time at the Mental Health Hospital in Al-Hassa, Kingdom of Saudi Arabia. Eligible male and female patients (*n* = 200) were approached, with 168 (84%) agreeing to participate in the study. The Internal Review Board of King Fahad Hospital-Hofuf in Saudi Arabia granted ethical approval for this study, and all the protocols were followed. Patients of both sexes aged 19 and above, psychiatric patients (Psych-P), and hospitalized and non-hospitalized patients were all eligible. Conditions for exclusion included declining to provide every pertinent detail, refusing to provide informed written permission for study participation from the participant or their legal representative, intellectual or physical deterioration that could hinder the collection of all important information, and the participant’s inability to participate fully in the research. However, if a relative agreed to offer information on their behalf, the patient could be included.

To detect a median-sized main effect with a statistical power of 1 − *β* = 0.90 (i.e., if the effect exists, there is a 90% chance of detecting a true positive), a two-tailed test with an α of 0.05 (i.e., there is a 5% risk of discovering a false positive) was used. To determine the required N, the three main parameters (anticipated d, target power, and *α*) were entered into G*Power, which utilized the following formula [25]:

$$N=\frac{4\times (\;Z_{1-\alpha /2=0.025}+Z_{1-\beta \;=0.90})2}{d_{(condition)2}}$$

where $\;Z_{1-\alpha /2=0.025}$ = 1.96 and $Z_{1-\beta =0.90\;}$= 1.28 are the critical *Z* values associated with a two-tailed test with *α* = 0.05 and 1 − *β* = 0.90, *d* = 0.50, respectively. We simply assumed that the intervention worked as expected, and a sample size of 168 participants gave us a 90% probability of observing the median-sized (or larger) effect of the condition with *p* < 0.05.

### 2.2. Sociodemographic Characteristic Data Collection

The data were acquired using a structurally verified questionnaire. The questionnaire was created to investigate the sociodemographic features of patients. Patients who could answer the questions were allowed to do so, otherwise, a family member was provided with an overview of the study and instructions on how to complete the questionnaire truthfully.

### 2.3. Anthropometric Measurement

The anthropometric parameters were measured using the AC-CUNIQ BC360 (SELVAS Healthcare Inc., Daejeon, Korea) body composition analyzer. Patients were asked to stand while electrodes were placed on their hands and feet to obtain measurements. The body mass index (BMI), waist circumference (WC), and body weight were automatically calculated when the device was connected to an ultrasonic height meter. Following each measurement, the patients’ palms and soles were cleaned, and they were told to fast for four hours before the test. The participants were allowed privacy during the testing. According to the WHO [26], a person’s body mass index falls into one of four categories: underweight (<18.5), normal (18.5–24.9), overweight (25–29.9), and obese (>30). In this study, three categories of obesity were considered: BMI ≥ 40 (severe obesity), BMI = 35–39.9 (Grade 2), and BMI = 30–34.9 (Grade 1). The waist circumference [27] was divided into three categories: extremely high weight (≥102 men, ≥88 women), high weight (94–101.9 men, 80–87.9 women), and normal weight (<94 males, <80 females).

### 2.4. Hematological and Biochemical Parameters

The XN1000-Cobas E411 (Sysmex-Rauch-Hitachi, Tokyo, Japan) was used to analyze patients’ blood and biochemical parameters in the laboratories of the Mental Health Hospital in Al-Hassa, Kingdom of Saudi Arabia. A fasting (before the first meal) venous blood sample was collected, and hematological and biochemical parameters were determined.

### 2.5. Statistical Method

The statistical program SPSS (software version 28.1; SPSS Inc., Chicago, IL, USA) was used to analyze the data. Frequencies and percentages are used to present the results. To evaluate the association between patient demographics and the classification of patients using fundamental hematological parameters, the chi-square test was employed. The predetermined levels of statistical significance were ** *p* ≤ 0.01 and * *p* ≤ 0.05. The study employed Spearman correlation coefficients and simple regression analysis to investigate the associations among demographic parameters, anthropometric proxies (BMI and WC), and depression type. The patient’s hematocrit (HCT), hemoglobin (Hb), and red blood cell count (RBC) were subjected to multiple regression analyses.

## 3. Results

### 3.1. Sociodemographic Characteristics of Patients

Table 1 displays the sociodemographic characteristics of the males and females with psychiatric diseases. Eligible male and female patients (*n* = 200) were approached, with 168 (84%) agreeing to participate in this study based on G-power analysis. The participants’ ages ranged between 19 and 59 years. Most of the 168 participants were females (30.34%) and males (27.85%) between the ages of 30 and 39. Furthermore, 68.35% of males and 51.69% of females were single. The majority of males (36.71%) were uneducated, whereas the majority of females (49.44%) were school-educated. The majority of males (55.70%) and females (60.67%) were unemployed. However, 74.68% of males and 80.90% of females reported a monthly income of SAR ≤ 5000. The majority of males (54.43%) and females (65.17%) were inpatients (hospitalized). Furthermore, the majority of males (46.84%) were diagnosed with schizophrenia spectrum and other psychotic disorders (SsP-d), while most females (44.94%) were diagnosed with depressive disorders (Dep-d).

### 3.2. Anthropometric Indices of Patients

Table 2 displays the body mass index (BMI) and waist circumference (WC) of males and females with psychiatric disorders. According to the BMI data, 25.32% of men and 30.34% of women were normal, while 1.27% of men and 10.11% of women were underweight. Approximately 32.91% of men and 21.35% of women were overweight. Furthermore, 17.98% and 20.22% of females, and 24.05% and 16.45% of males, suffered from obesity (I) and obesity (II and III), respectively, and other related conditions. Furthermore, 16.46% of males and 20.23% of females were revealed to have a high WC, 65.82% of males and 38.20% of females had a normal WC, and 17.72% of males and 41.57% of females had a very high WC.

### 3.3. Basic Hematological Parameter Levels of Patients

To investigate the health consequences, the basic hematological and biochemical parameter levels of patients were determined. Table 3 displays the fundamental parameter levels of male and female patients. The frequency distributions of hematological and biochemical marker levels between male and female patients differed significantly. In terms of hemoglobin (Hb) levels, 59.49% of males had normal levels and 40.51% had low levels, while 50.56% of females had normal levels and 49.44% had low levels. The majority of males (60.76%) and females (58.43%) had significantly reduced hematocrit (HCT) levels. Females (69.66%) had a lower mean corpuscular hemoglobin concentration (MCHC) than males (46.84%), whereas blood urea nitrogen (BUN) levels were normal in 61.80% of females and 48.10% of males. Creatine kinase (CK) levels were normal in most males (63.29%) but higher in most females (55.06%), while red cell distribution width–standard deviation (RDW-SD) levels were lower in most males (93.67%) and females (80.90%), as were the levels of vitamin B12 (57.0% and 95.51%, respectively) and vitamin D3 (91.14% and 100%, respectively). The procalcitonin test (PCT) was found to be lower in 51.90% of males but normal in 69.66% of females. However, the majority of the hematological and biochemical markers in both non-anemic sexes were determined to be normal, with anemic patients having lower levels, especially of Hb and vitamin B12.

**Figure 1:**
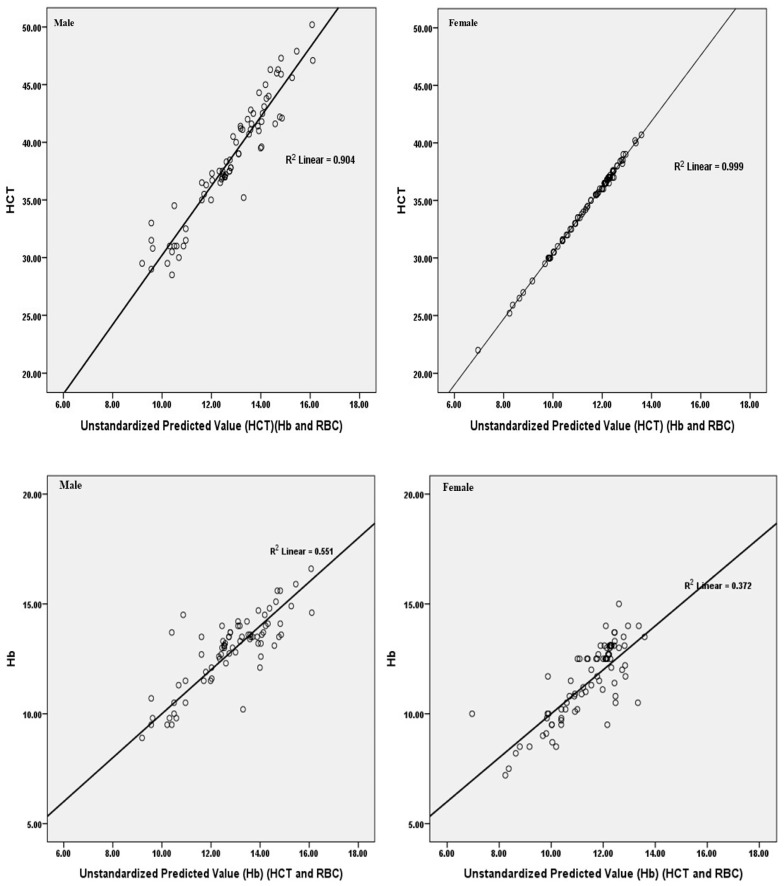
Multiple regression analyses between hematocrit (HCT) and both hemoglobin (Hb) and red blood cell count (RBC). For males, R^2^ = 0.904; for females, R^2^ = 0.999; and between Hb and both HCT and RBC for males, R^2^ = 0.551; and for females, R^2^ = 0.372.

The multiple regression analysis, as shown in Figure 1, revealed that hemoglobin (Hb) and red blood cell count (RBC) explained 90.4% (R^2^) of the variance in hematocrit (HCT) for males and 99.9% (R^2^) for females. Furthermore, hematocrit and red blood cell count explained 55.1% (R^2^) of the variance in hemoglobin in males, but only 37.2% (R^2^) in females.

### 3.4. Categorization of Psychiatric Disorders and Hematological Markers Among Patients

Table 4 categorizes males and females with psychiatric disorders based on hematological and biochemical markers and the psychologists’ diagnosis of depression. According to the diagnosis, 26.58% of males and 44.94% of females had depressive disorders (Dep-d), 46.83% of males and 29.21% of females had schizophrenia spectrum and other psychotic disorders (SsP-d), 6.33% of males and 4.49% of females had personality disorders (Pd), 5.06% of males and no females were diagnosed with neurodevelopmental disorders (Nero-d), and 7.59% of males and 14.61% of females had bipolar and related disorders (Bip-d). Based on the hematological and biochemical parameter values, anemia was found in 40.51% of the male patients and 49.44% of the female patients with psychotic conditions who took part in the research. Within the anemic groups, 57.14% had depressive disorders (Dep-d), 32.43% had psychotic disorders (SsP-d), 40% had personality disorders (Pd), 25% had neurodevelopmental disorders (Nero-d), 66.67% had bipolar and related disorders (Bip-d), 20% had anxiety disorders (Ad), and none of those with disorders related to trauma and stressors (TSR-d) had anemia. Anemia was found in 49.44% of female patients with psychotic disorders, divided into 22% with depressive disorders and 25% with bipolar disorders. According to the hematological and biochemical markers and the psychologist’s diagnosis of depression, both male and female patients had marker levels below normal, with few markers falling within the range, but some markers, such as creatinine and creatinine kinase in serum, were above normal.

### 3.5. Correlation of Sociodemographic Characteristics, Anthropometric Indices, and Type of Depression

Table 5 lists the correlation between sociodemographic characteristics, anthropometric indices, and type of depression for patients with psychiatric disorders, as determined by the Spearman correlation coefficient and simple regression analysis. BMI and WC were utilized to assess the patient’s anthropometric indices. The relationships between age and BMI and WC were significant (*p* ≤ 0.05) and positively correlated in both males (*β* * = 0.103 *, 0.01) and females (*β* * = 0.390 *, 0.32). However, for both sexes, education level was dramatically (*p* ≤ 0.001) and adversely linked with BMI and WC, with a strong influence on WC for males (*β* ** = −0.128 **, 0.12) and BMI for females (*β* ** = −0.204 **, 0.36). Conversely, for males only, marital status was positively and strongly (*p* ≤ 0.001) linked with BMI and WC, with a large influence on WC (*β* ** = 0.115 **, 0.14). Furthermore, only the work status and the WC of males showed a significant (*p* ≤ 0.05) positive link (β** = 0.013 *, 0.07). Male income per month was discovered to be significantly (*p* ≤ 0.05) positively associated with both BMI and WC, with a significant impact on BMI (*β* * = 0.110 *, 0.13), and inversely linked with both BMI and WC, with a high impact on WC (*β* * 8 = −0.018 **, 0.166), but neither had an impact on the indices for females. The gender-specific diagnosis had a significant negative connection with both BMI and WC, with a high impact on BMI for males (*β* * = −0.118 *, 0.03) and females (*β* ** = −0.107 **, 0.15). As shown in Table 5, there is a significant and affirmative association between the kind of depression and the age of both males (*p* ≤ 0.001) and females (*p* ≤ 0.05). Nonetheless, there was a favorable correlation between the male type of depression and their marital status. Both male and female depression types were substantially (at either *p* ≤ 0.05 or *p* ≤ 0.001) adversely correlated with employment, education, and monthly income.

## 4. Discussion

The current study investigated the relationship between sociodemographic characteristics, anthropometric indices, and psychological disorders among patients in Saudi Arabia’s Eastern Region (Al-Hassa), as well as their health consequences, including basic hematological and biochemical markers. Males and females were found to have different sociodemographic characteristics. Patients’ ages varied considerably both within and between the sexes, with a high prevalence of individuals between the ages of 30 and 39. According to these data, there was a vital age range within which both males and females could experience long-term mental health problems. The age frequency of major depressive disorders was substantially linked with family structure and socioenvironmental characteristics in the patient’s family of origin [28]. However, Joinson et al. [29] observed that, across all age categories, low socioeconomic status was associated with increased incidence rates of depressive symptoms; the strongest relationships were identified with indicators of low quality of living. There was no evidence that the age at which symptoms manifested could be used to subtype depressive symptoms because there was a strong association between low socioeconomic status and both early- and later-onset symptoms. Even though married respondents’ percentages were much lower than those of single participants, all of them suffered from a persistent mental health disorder. Self-reported measures of marital dissatisfaction and depressive symptoms were significantly linked to community samples for both males and females [30]. Males made up a large percentage of the illiterate participants, while females made up the majority with a school degree. These findings suggest that education significantly influenced the occurrence of depression symptoms. According to the present study’s findings, participants who completed only their primary schooling had a higher chance of being depressed than those who attended college or university. These results align with those of Alhomoud et al. [31], who demonstrated that sickle cell anemia patients with lower than a high school degree were more likely to experience depression. The majority of patients had low incomes and no jobs, which is likely one of the main causes of their chronic mental illnesses. Furthermore, compared to patients with high monthly incomes (SAR > 5000), those with low monthly incomes (SAR ≤ 5000) have almost twice as high a chance of developing depression. A similar result was observed by Alhomoud et al. [31] in sickle cell anemia patients, who may be unable to keep or maintain regular jobs due to their poor educational level, the chronic nature of their illness, and their frequent hospitalizations. Additionally, these results concur with the findings of Hakulinen et al. [32], who found that in the years before their initial hospitalization, people with serious mental illnesses had lower total incomes and were less able to work. The majority of patients required intensive care, as seen by the increased percentage of hospitalized patients in the results. According to Duran-Badillo et al. [33], functional dependence increased with depression in older persons; similarly, higher functional dependency was associated with anxiety and lower cognitive performance. As reported by Blázquez et al. [34], diagnostic stability is crucial for the acceptance of mental illnesses and provides a foundation for forecasting their trajectory and result. In addition, DeDoménico et al. [35] reported that establishing a precise diagnosis in psychiatry is crucial for the development of suitable and effective treatments for every patient. According to research by Duran-Badillo et al. [33], the functional dependency of older hospitalized patients is impacted by several variables, including age, gender, marital status, cognitive function, depression, and anxiety. Furthermore, Kohn et al. [36] noted that psychiatric patients may not receive proper medical care due to a variety of factors, including the failure of mental health professionals to recognize physical symptoms, the diagnosis of physical complaints as psychosomatic, a lack of time and resources for screening in mental health services, the patient’s inability to effectively explain their medical problems, and challenges implementing lifestyle changes. Agerbo et al. [37] stated that the first category of risk factors for depression generally includes socioeconomic risk factors such as sex (more prevalent in women), low income, low education, noneconomic activity, divorce and bereavement, smoking and nicotine-dependence symptoms, alcohol consumption, ethnic group, and occupation level.

The data in this study demonstrated that most of the anthropometric proxies were greater in depressed males than in females, particularly those who were overweight or obese. In this regard, Grzymisławska et al. [38] found that gender influences nutritional behavior, styles of nutrition, dietary profiles, attitude to nourishment, place of meal intake, and nutritional knowledge sources. Furthermore, gender differences in nutritional intake and appetite control indicate that females are more readily satiated than males, due in part to gender-specific hormonal and neural activity [39]. Furthermore, they reported that dietary patterns also show gender differences; females prefer healthier options such as vegetables, whereas males consume more milk, fermented items, and carbohydrates. Psychologically, males are more extroverted, whereas females are more conscientious, open, and prone to negative thoughts and anxieties. In terms of physical activity, females train more frequently than males [40]. It has been shown that genetic variables influence the chance of being obese [41]. Kim et al. [42] found that high genetic risk and an obesogenic lifestyle were both separately and together related to an increased risk of obesity and differed between males and females. The result of this study agrees with an investigation that observed an increase in depression related to waist circumference, BMI, and obesity, particularly in males [43]. This may be explained by the dietary practice of depressed males who consume more fatty foods, which causes visceral and abdominal obesity and, in turn, triggers systemic inflammation and disorders [43]. According to Geiker et al. [44], adults who are under constant stress are more likely to be obese and have a larger waist circumference than adults who are not under stress. Stress has been shown to impact changes in weight through physiological processes, dietary habits, levels of physical activity, sleep patterns, and sedentary behavior. Psychotic disorder patients live in unfavorable environments where they are unable to express themselves and cannot satisfy even their most basic needs for housing and food [45]. Generally, and according to Blasco et al. [46], anthropometric characteristics associated with obesity, such as weight, waist circumference, and body mass index, are typically included in the second group of depression.

To explore the health status of the patients, basic hematological and biochemical parameter levels were measured. Male and female patients exhibited considerably differing levels of basic hematological indicators. A subset of the markers, including serum creatinine and creatinine kinase, were above normal in both male and female patients, while other markers were below or within the normal range. As shown, hematocrit (HCT) is the volume percentage of red blood cells (RBCs) in total blood as well as hemoglobin (Hb), and a low HCT level is connected with low RBC and Hb levels, potentially increasing the risk of cardiovascular disease, especially in males. A study by Akaishi et al. [19] found that reduced hemoglobin (Hb) levels and raised neutrophil-to-lymphocyte, monocyte-to-lymphocyte, and platelet-to-lymphocyte ratios had a significant effect on depression symptoms and sleep disruptions in the general population. These relationships remained significant even after accounting for potential variables such as age, gender, BMI, and daily tobacco use. Both males and females showed associations, although males showed them more prominently. The precise association between circulating blood cell patterns and mental health issues such as depression and sleep disorders in the general population remains unknown. Low hemoglobin levels have been found to have a deleterious impact on a wide spectrum of psychiatric diseases such as depression and impaired cognitive abilities [21,22]. Many factors influence hemoglobin levels, including sex, age, and smoking status [23]. Vitamin B12 deficiency and folate deficiency anemias are conditions in which not enough healthy red blood cells are created to transport oxygen throughout the body, and their absence can cause several illnesses [24]. Yildiz et al. [47] found that seriously depressed patients had significantly higher plasma viscosity, a lower erythrocyte elongation index, and lower mean corpuscular volume, mean corpuscular hemoglobin, and mean corpuscular hemoglobin concentration values. Platelets, total cholesterol, triglycerides, high-density lipoprotein cholesterol, red and white blood cells, hemoglobin, hematocrit, glucose, alanine transaminase, and blood urea nitrogen are generally considered to be biochemical factors linked to depression [48,49].

Based on hematological and biochemical characteristics as well as psychological evaluations, the majority of male patients were diagnosed with schizophrenia spectrum and other psychotic disorders (SsP-d), while the majority of female patients were diagnosed with depressive disorders (Dep-d). Brivio et al. [50] stated that the gender disparity in depression is one of the most important phenomena in psychiatry and psychology, with more females than males reporting depression. For instance, females are more prone than males to experience changes in metabolism and sleep habits as well as more severe symptoms when suffering from depression. Based on the levels of hematological and biochemical indicators, anemia was detected in 40.51% of the males who participated in this study, with a higher percentage among those who experienced depressive disorders (Dep-d) and schizophrenia spectrum and other psychotic disorders (SsP-d), and in 49.44% of females who experienced depressive disorders (Dep-d). Hb and RBC accounted for 90.4% and 99.9% of the variance in HCT for males and females, respectively, whereas HCT and RBC explained 55.1% and 37.2% of the variance in Hb for males and females, respectively. Based on the findings, we hypothesized that anemia is more common in people from lower socioeconomic backgrounds who have limited access to healthcare and struggle to communicate their medical concerns. Consequently, it makes sense that the anemia rate among mental health patients would be higher than that of the general population. The outcomes of this investigation, however, were found to be better than those of other published studies. Anemia is known to occur more frequently in women of reproductive age who lose blood from menstrual bleeding [51]. Our statistics support this by demonstrating that a greater proportion of females than males were anemic. An increased risk of adult depression was linked to anemia, according to a meta-analysis of observational epidemiological research [52]. This observed correlation could perhaps stem from susceptibility characteristics shared by depression and anemia. Patients with psychotic disorders live in unfavorable environments where they are unable to express themselves and cannot satisfy even their most basic needs for housing and food [45]. This may cause shortages in iron and zinc as well as vitamins B12 and folic acid, which could result in anemia [52]. Low blood ferritin and Hb levels were associated with depression in a study involving young adult males and females [53].

To confirm the relationship between sociodemographic variables, anthropometric indices, and psychiatric illnesses among patients, the Spearman correlation coefficient and simple regression analysis of such variables was used. The findings demonstrated that the majority of sociodemographic factors were either positively or negatively connected to patients’ anthropometrics and the type of depression in both sexes. The current investigation indicates that the patterns of overweight and obesity in both sexes worsen with an increase in age. Okati-Aliabad et al. [54] noted that people in Middle Eastern countries have a growing susceptibility to obesity with age, and there is a significant frequency of obesity and overweight among people over 40. Physiological reasons, including weight gain after menopause, the corresponding drop in metabolic expenditure, and the aging-related decline in physical activity, can all explain this link. However, as evidenced by Bakir et al. [55], obese adults are more likely to experience serious health consequences, making the age-related rise in obesity an important issue. In a study by Al-Ghamdi et al. [56], a linear and positive trend was shown between growing older and having a high BMI for both genders. The BMI also gradually increased over adulthood, peaking at 50–59 years for both genders. Males with a significantly high BMI and WC were observed to gain weight after marriage, according to the current results. However, there was no discernible link between a female’s marital status and her BMI or WC. According to Liu et al. [57], married individuals had a 2.5-fold higher likelihood of being obese than single, divorced, or widowed individuals, and men were more likely than women to be overweight or obese. Furthermore, they showed that the chances of being overweight or obese were similar for married individuals and those who had divorced or had been widowed.

Bakir et al. [55] found that married women have a greater probability of being overweight or obese, and their high BMI may be caused by a decrease in exercise, a higher parity, or dietary patterns that show an increase in food intake after marriage. In the current study, there was an inverse relationship between education and BMI and WC for both genders. Our findings have been confirmed by other investigations. For instance, Witkam et al. [58] found that higher levels of education in both men and women were linked to a decreased risk of obesity. They also concluded that gender and the degree of national economic development affected the relationship between obesity and educational attainment, with higher-income countries showing more powerful social patterning in the direct association and lower-income countries showing a negative association. Education has also been linked to a high incidence of obesity and overweight in both sexes [59]. It was found that, whereas female WC and BMI were not significantly impacted by these factors, monthly income and work status were strongly connected with male anthropometric indices. An earlier study [56] determined a correlation between a higher BMI and employment as well as a higher monthly income. According to an investigation by Harkko et al. [60], stress, depression, and anxiety caused by unfavorable economic circumstances can have physiological impacts, including depression of the immune system, which affects cardiovascular health, while unemployment predisposes both sexes to poor mental health. An increased risk of depression and cardiovascular diseases was linked to unemployment [61]. Furthermore, losing one’s job is linked to the start or worsening of bad behaviors such as smoking and excessive drinking. On the other hand, women’s unemployment (but not men’s) is strongly linked to weight gain. This can be explained by the fact that working females are often young, in addition to the fact that their exposure to the community at work encourages them to maintain a healthy weight [54]. According to Ribeiro et al. [62], there may be a connection between mental health issues and wealth inequality, which is supported by the data in this investigation. If there is a causal link between these two variables, then rising income inequality may raise the prevalence of mental health issues, and its decline may significantly improve population well-being. Male anthropometric indices but not female anthropometric indices were inversely associated with the state of the patients, according to the association between the two variables. This suggests that outpatient management does not exist. Accordingly, research indicates that a sizable fraction of outpatients experience depression, emphasizing the significance of creating efficient management plans for the prompt detection and treatment of these disorders in outpatients in clinical practice [63]. All sorts of diagnoses (psychotic disorders) were observed to influence anthropometric indices in both males and females. We discovered that the link between weight and depression symptoms did not differ significantly between males and females. Males but not females showed a reverse link between depressed symptoms and body weight when Tronieri et al. [64] examined the relationship between depression and body weight in different sexes with different ages. Another study suggested that depression is more widespread among obese persons, particularly females, despite some data relating obesity to reduced levels of depressive symptoms and anxiety [65]. Obesity is more likely to be connected with depression onset in men than in women. However, regardless of whether a person is underweight or overweight, the link between weight and depression symptoms is negative for both men and women [66]. The findings revealed that as people aged, both men and women experienced various forms of depression. On the other hand, marital status was found to increase male depression. Low levels of education, unemployment, and poor monthly income were observed to enhance the prevalence of all types of depression in both males and females. The prevalence of any psychiatric disease decreased with age in males but increased in females [67].

According to another study, depression may have a different correlation with patients’ educational attainment depending on their gender, age, and race [68]. Individuals who are unsatisfied with their relationships are more likely to experience clinical depression than those who are satisfied. Marital discontent may not only raise one’s susceptibility to depression, but it may also hasten its progression and hinder recovery [31]. Qin et al. [69] discovered significant socioeconomic gradients in mental health: higher levels of education and income are associated with a lower likelihood of depression, especially among individuals with a lower socioeconomic status. Liu et al. [70] studied depression symptoms and their association with social factors and chronic diseases in middle-aged and elderly Chinese adults, discovering that age, education, and income were all linked to depressive symptoms. These findings coincide with a previous study that indicated low employment rates among patients with schizophrenia and other non-affective psychoses [71]. Employment is frequently viewed as a recovery aim for people suffering from serious mental illnesses. Assistance with employment and the individual placement and support (IPS) model is at least twice as effective as other programs in placing people with severe mental illnesses in competitive jobs [72]. According to Ryu and Fan [73], the relationship between financial problems and psychological discomfort was stronger among the unmarried, the unemployed, low-income households, and renters than among their counterparts. Furthermore, Walker et al. [74] reported that depression and anxious symptoms were most commonly associated with advanced disease, low levels of education, and low income.

## 5. Limitations

Challenges were encountered in effectively communicating with some patients regarding their participation in the study. Because this study was cross-sectional, the results must be treated with caution. Data collection on physical activity was challenging, as many participants reported low levels of physical activity, which may reflect common lifestyle patterns within the study population. Finally, the sample size was small owing to the limited number of accessible cases because we focused on a specific location in Saudi Arabia; covering the entire Kingdom of Saudi Arabia in the study was impossible due to the country’s size. The limitations of the current investigation will be addressed in future studies by increasing the sample size to include both public and private hospitals and comparing the prevalence of MS among large groups of males and females with that among healthy participants.

## 6. Conclusions

This study determined that the majority of males and females with psychotic disorders had low sociodemographic characteristics associated with high anthropometric indices and depression type, which were greater in depressed males than in females, particularly those who were overweight or obese. Furthermore, a poor lifestyle and a low education level correlate with obesity, which may lead to depression and result in low levels of hematological and biochemical parameters in both male and female patients. In addition, the results revealed that psychotic disorders differed between males and females. As a result, age, education, marital status, employment, and monthly income might be considered risk factors for individuals with psychotic disorders and have been shown to have an impact on their health. The findings suggest that both medical personnel and health authorities should pay attention to the sex disparities in psychotic disorder-related components. We believe that the most successful treatment method for psychotic disorders is lifestyle adjustment, with a focus on maintaining a healthy weight and engaging in regular physical activity. Overall, patients with psychotic disorders should be encouraged to make lifestyle changes such as incorporating frequent exercise, eating a healthy diet, and quitting smoking. Public health data and behavioral patterns clearly show that a lack of awareness is a key contributor to obesity; thus, it is critical to conduct community health efforts aimed at reducing the obesity epidemic. In the future, it is recommended that herbal medicine interventions for obesity and overweight in such groups be standardized.

## Figures and Tables

**Table 1:** Sociodemographic characteristics of chronic psychiatric patients (*n* = 168).

Demographic Characteristics	Male			Female			Chi-Square Between Sexes
	Frequency	%	Chi-Square	Frequency	%	Chi-Square	
Age							
19–29	20	25.32	9.67 * p = 0.046	24	26.97	18.58 ** p = 0.001	27.12 ** p < 0.001
30–39	22	27.85		27	30.34		
40–49	16	20.25		21	23.59		
50–59	15	18.99		12	13.48		
>59	6	7.59		5	5.62		
Total	79	100.0		89	100.0		
Marital Status							
Single	54	68.35	10.65 **	46	51.69	0.101	6.09 * p = 0.013
Married	25	31.65	*p* = 0.001	43	48.31	0.750	
Total	79	100.0		89	100.0		
Education							
Uneducated	29	36.71	0.481 0.786	11	12.36	19.30 ** p = 0.006	8.14 * p = 0.017
School education	26	32.91		44	49.44		
University or college	24	30.38		34	38.20		
Total	79	100.0		89	100.0		
Employment status							
Not employed	44	55.70	1.03	54	60.67	4.06 *	4.67 * p = 0.031
Employed	35	44.30	*p* = 0.311	35	39.33	*p* = 0.044	
Total	79	100.0		89	100.0		
Monthly income							
SAR ≤ 5000	59	74.68	19.25 **	72	80.90	33.98 **	52.59 ** p = 0.001
SAR > 5000	20	25.32	*p* < 0.001	17	19.10	*p* = 0.001	
Total	79	100.0		89	100.0		
Patient status							
Outpatient (Unhospitalized)	36	45.57	0.620	31	34.83	8.19 **	6.88 ** p = 0.009
Inpatient (Hospitalized)	43	54.43	*p* = 0.431	58	65.17	*p* = 0.004	
Total	79	100.0		89	100.0		
Type of diagnosis							
Depressive disorders (Dep-d)	21	26.58		40	44.94	78.66 ** p < 0.001	175.83 ** p < 0.001
Schizophrenia spectrum and other psychotic disorders (SsP-d)	37	46.84		26	29.21		
Personality disorders (Pd)	5	6.33	90.51 ** p < 0.001	4	4.49		
Neurodevelopmental disorders (Nero-d)	4	5.06		0	0.00		
Bipolar and related disorders (Bip-d)	6	7.59		13	14.61		
Anxiety disorders (Ad)	5	6.33		5	5.62		
Trauma- and stressor-related disorders (TSR-d)	1	1.27		1	1.13		
Total	79	100.0		89	100.0		

Chi-square = (* *p* ≤ 0.05, ** *p* ≤ 0.01).

**Table 2:** Body mass index (BMI) and waist circumference of males and females with chronic psychiatric disorders (*n* = 168).

Anthropometric Index	Males		Females	
	F (%)	Mean ± SD	F (%)	Mean ± SD
Body mass index (BMI)				
Underweight (<18.5)	1 (1.27)	__	9 (10.11)	16.94 ± 0.931
Normal (18.5–24.9)	20 (25.32)	22.82 ± 1.60	27 (30.34)	20.94 ± 1.88
Overweight (25–29.9)	26 (32.91)	27.15 ± 1.09	19 (21.35)	28.11 ± 1.28
Obesity I (30.0–34.9)	19 (24.05)	32.84 ± 1.51	16 (17.98)	32.31 ± 1.26
Obesity II, III (≥35)	13 (16.45)	39.16 ± 3.47	18 (20.22)	41.89 ± 6.97
Total	79 (100.0)	29.29 ± 6.04	89 (100.0)	28.35 ± 9.04
Waist circumference				
Normal (<94 men, <80 women)	52 (65.82)	84.21 ± 6.65	34 (38.20)	70.40 ± 10.88
High (94–101.9 men, 80–87.9 women)	13 (16.46)	98.23 ± 2.24	18 (20.23)	84.22 ± 2.07
Very high (≥102 men, ≥88 women)	14 (17.72)	107.50 ± 3.57	37 (41.57)	101.43 ± 12.70
Total	79 (100.0)	90.65 ± 10.96	89 (100.0)	86.09 ± 17.49

BMI: chi-square = 35.373 (*p* value = 00.001). Waist circumstance: chi-square: 17.765 = (*p* = 0.0013).

**Table 3:** Frequency distribution of males and females with chronic psychiatric disorders according to basic hematological and biochemical parameter levels (*n* = 168).

Variables	Male (*n* = 79)			Chi-Square	Female (*n* = 89)			Chi-Square	Chi-Square Between Sexes
	Low	Normal	High		Low	Normal	High		
	F (%)	F (%)	F (%)		F (%)	F (%)	F (%)		
Hemoglobin (Hb, g/dL)	32 (40.51)	47 (59.49)	__	2.85 *	44 (49.44)	45 (50.56)	__	0.011	3.43 *
Hematocrit (HCT, %)	48 (60.76)	31 (39.24)	__	3.66 *	37 (41.57)	52 (58.43)	__	2.53	0.024
Red blood cell count (RBC, 10^6^ cells/mcL)	21 (26.58)	51 (64.56)	7 (8.86)	38.38 **	18(20.22)	69 (77.53)	2 (2.25)	82.54 **	117.75 **
Erythrocyte range (ER, 10^6^ cells/mcL)	15 (19.0)	64 (81.0)	__	30.39 **	4 (4.49)	82 (92.14)	3 (3.37)	138.49 **	219.25 **
Mean corpuscular volume (MCV, fL)	16 (20.25)	63 (79.75)	__	27.96 **	15 (16.85)	74 (83.15)	__	39.11 **	66.88 **
Mean corpuscular hemoglobin (MCH, pg/cell)	33 (41.77)	45 (56.96)	1 (1.27)	39.29 **	36 (40.45)	51 (57.30)	2 (2.25)	42.49 **	81.75 **
Mean corpuscular hemoglobin concentration (MCHC, g/dL)	37 (46.84)	42 (53.16)	__	0.316	62 (69.66)	27 (30.34)	__	13.76 **	5.36 *
Blood urea nitrogen (BUN, mg/dL)	30 (37.98)	38 (48.10)	11 (13.92)	14.61 **	16 (17.98)	55 (61.80)	18 (20.23)	32.52 **	39.25 **
Platelet count (PLT, platelets/mcL)	3 (3.80)	76 (96.20)	__	67.46 **	1 (1.12)	88 (98.88)	__	85.05	152.38 *
Creatinine in serum (CS, mg/dL)	__	36 (45.57)	43 (54.43)	0.620	__	64 (71.91)	25 (28.09)	17.09 **	6.09 *
Creatine Kinase (CK, U/L)	__	50 (63.29)	29 (36.71)	5.58 *	__	40 (44.94)	49 (55.06)	0.910	0.857
Red cell distribution width–standard deviation (RDW-SD, fL)	74 (93.67)	5 (6.33)	__	60.27 **	72 (80.90)	17 (19.10)	__	33.99 **	91.52 **
Platelet distribution width (PDW, fL)	__	78 (98.73)	1 (1.27)	75.05 **	1 (1.12)	84 (94.38)	4 (4.50)	149.42 **	301.10 **
Mean platelet volume (MPV, fL)	9 (11.39)	70 (88.61)	__	47.10 **	1 (1.12)	86 (96.63)	2 (2.25)	160.47 **	268.42 **
Platelet–large cell ratio (P-LCR, fL)	1 (1.27)	78 (98.73)	__	75.05 **	1 (1.12)	88 (98.88)	__	85.05 **	160.09 **
Procalcitonin test (PCT, %)	41 (51.90)	38 (48.10)	__	0.114	27 (30.34)	62 (69.66)	__	13.76 **	6.09 *
Vitamin B12 (pg/mL)	45 (57.0)	34 (43.0)	__	1.53	85 (95.51)	4 (4.49)	__	73.72	50.38 **
Vitamin D3 (ng/mL)	72 (91.14)	6 (7.59)	1 (1.27)	119.27 **	89 (100.0)	__	__	__	295.53 **

** *p* ≤ 0.01; * *p* ≤ 0.05. According to the instrument manual: Hb, normal = 13.0–17.5 for males, normal = 12–15.5 for females; HCT, normal = 40–52% for males, normal = 35–47% for females; RBC, normal = 4.3–5.9 for males, normal = 3.5–5.5 for females; ER, normal = 4.3–5.9 for males, normal = 3.5–5.5 for females; MCV, low < 80, normal 80–100, high > 100 for males and females; MCH, low < 27, normal 27–31, high > 31 for males and females; MCHC, low < 32, normal 32–36, high > 36 for males and females; BUN, low < 7, normal 7–20, high > 20 for males and females; PLT, low < 150, normal 150–450, high > 450 for males and females; CS, normal = 0.6–1.3 for males, normal = 0.5–1.1 for females; CK, low < 22, normal 22–198, high > 198 for males and females; RDW-SD, low < 39, normal 39–46, high > 46 for males and females; PDW, low < 9.5, normal 9.5–17.5, high > 17.5 for males and females; MPV, low < 7.5, normal 7.5–11.5, high > 11.5 for males and females; P-LCR, low < 13.5, normal 13.5–43.5, high > 43.5 for males and females; PCT, low < 0.2, normal 0.2–0.5, high > 0.5 for males and females; vitamin B12, low < 200, normal 200–900, high > 900 for males and females; vitamin D3, low< 30, normal 30–100, high > 100.

**Table 4:** Categorization of psychiatric disorders and anemia among males and females based on hematological and biochemical parameters.

Parameters	Male—Anemia (*n* = 32)							Female—Anemia (*n* = 44)						
	Dep-d *n* = 21	SsP-d N = 37	Pd *n* = 5	Nero-d *n* = 4	Bip-d N = 6	Ad *n* = 5	TSR-d *n* = 1	Dep-d *n* = 40	SsP-d *n* = 26	Pd *n* = 4	Nero-d *n* = 0	Bip-d *n* = 13	Ad *n* = 5	TSR-d *n* = 1
Anemia, *n* (%)	12 (57.14)	12 (32.43)	2 (40)	1 (25)	4 (66.67)	1 (20)	_	21 (52.50)	12 (46.15)	3 (75.0)	_	5 (38.46)	2 (40.0)	1 (100)
Hb (g/dL)	10.29	12.21	12.15	11.60	8.93	10.50	_	9.75	10.26	9.03	_	9.96	11.25	9.80
HCT (%)	31.69	37.72	33.50	36.70	34.20	32.50	_	32.05	32.72	30.73	_	33.78	34.25	31.60
RBC (10^6^ cells/mcL)	3.60	4.81	4.50	4.31	3.87	4.00	_	3.83	4.31	2.83	_	3.96	4.10	3.99
ER (10^6^ cells/mcL)	4.10	4.41	4.65	4.39	4.20	4.20	_	5.31	4.68	4.13	_	4.48	4.65	5.21
MCV (fL)	85.27	80.83	87.25	85.00	84.50	80.00	_	83.86	79	75.33	_	80.30	83.25	79.20
MCH (pg/cell)	26.93	26.77	26.65	27.00	25.83	26.00	_	26.37	25.51	22.17	_	25.10	.27.50	
MCHC (g/dL)	31.07	31.72	33.50	31.70	30.45	32.50	_	29.22	30.12	25.93	_	30.16	32.15	31.00
BUN (mg/dL)	17.63	7.54	20.50	3.10	20.00	10.40	_	15.91	14.62	23.00	_	13.06	12.00	5.60
PLT (platelets/mcL)	169.08	221.13	167.50	418.00	169.75	175.00	_	170.43	221.17	168.33	_	183.20	175.00	314.00
CS (mg/dL)	1.09	56.87	1.05	32.62	0.90	1.10	_	1.20	20.77	12.63	_	18.22	1.90	70.00
CK (U/L)	245.00	197.33	139.00	233.00	163.75	95.00	_	281.05	202.93	433.33	_	187.60	310.00	250.00
RDW-SD (fL)	37.59	18.86	39.10	16.00	35.00	38.00	_	35.39	28.07	36.67	_	38.07	26.60	35.43
PDW (fL)	11.06	15.88	11.30	16.80	11.05	11.00	_	11.19	13.86	10.60	_	51.10	11.10	17.28
MPV (fL)	8.53	8.45	8.85	8.20	8.63	8.50	_	8.74	9.13	8.90	_	8.64	8.60	9.19
P-LCR (fL)	16.40	16.47	16.50	16.59	16.50	14.40	_	16.62	16.27	15.87	_	15.92	17.25	16.44
PCT (%)	0.258	0.186	0.350	0.190	0.300	0.300	_	0.271	0.277	0.200	_	0.266	0.300	0.25
Vitamin B12 (pg/mL)	149.58	195.00	180.00	210.00	162.50	140.00	_	150.95	128.67	103.33	_	157.60	147.50	150.00
Vitamin D3 (ng/mL)	13.13	22.21	20.50	30.00	18.08	18.00	_	15.54	16.48	9.27	_	14.60	11.75	13.00

Depressive disorders—Dep-d; schizophrenia spectrum and other psychotic disorders—SsP-d; personality disorders—Pd; neurodevelopmental disorders—Nero-d; bipolar and related disorders—Bip-d; anxiety disorders—Ad; Trauma- and stressor-related disorders—TSR-d.

**Table 5:** Spearman correlation coefficients between sociodemographic factors, anthropometric indices (BMI and WC), and type of depression for males and females with chronic psychiatric disorders (*n* = 168).

Dependent Variable/ Independent Variable	Male						Female					
	BMI			WC			BMI			WC		
	Rho	(*β*, r^2^)	*p*-Value	Rho	(*β*, r^2^)	*p*-Value	Rho	(*β*, r^2^)	*p*-Value	Rho	(*β*, r^2^)	*p*-Value
Age	0.110 *	(0.103 *, 0.01)	0.033	0.137 *	(0.182 *, 0.001)	0.028	0.569 *	(0.390 *, 0.32)	0.042	0.380 *	(0.111 *, 0.14)	0.023
Marital status	0.166 *	(0.117 *, 0.03)	0.044	0.369 **	(0.115 **, 0.14)	0.001	0.049	(0.004, 0.002)	0.651	0.101	(0.074, 0.01)	0.990
Education	−0.124 *	(−0.123 *, 0.02)	0.027	−0.349 **	(−0.128 **, 0.12)	0.002	−0.602 *	(−0.204 **, 0.36)	0.050	−0.441 *	(−0.232 **, 0.19)	0.038
Employment status	0.074	(0.009, 0.002)	0.518	0.256 *	(0.013 *, 0.07)	0.023	0.004	(0.002, 0.001)	0.173	0.071	(0.013, 0.03)	0.510
Monthly income	0.366 *	(0.110 *, 0.13)	0.050	0.300 **	(0.114 **, 0.09)	0.007	0.056	(0.001, 0.003)	0.605	0.069	(0.004, 0.005)	0.520
Patient status	−0.189 *	(−0.011 *, 0.04)	0.035	−0.394 **	(−0.018 **, 0.166)	0.001	−0.364	(−0.007, 0.13)	0.097	−0.404	(−0.002, 0.16)	0.090
Type of diagnosis	−0.173 *	(−0.118 *, 0.03)	0.021	−0.149 *	(−0.028 *, 0.02)	0.050	−0.358 **	(−0.107 **, 0.15)	0.006	−0.455 *	(−0.209 *, 0.21)	0.050
**Dependent Variable/** **Independent Variable**	**Male Type of Depression**			**Female Type of Depression**								
	**Rho**	**(*β*, r^2^)**	***p*-Value**	**Rho**	**(*β*, r^2^)**	***p*-Value**						
Age	0.214 **	(0.205 **, 0.046)	0.001	0.399 *	(0.275 *, 0.683)	0.027						
Marital status	−0.267 *	(−0.173 *, 0.071)	0.042	−0.129	(−0.164, 0.280)	0.068						
Education	−0.198 *	(−0.161 *, 0.039)	0.040	−0.323 *	(−0.305 *, 0.104)	0.010						
Employment status	−0.163 *	(−0.159 *, 0.027)	0.030	−0.130 **	(−0.238 **, 0.017)	0.002						
Monthly income	−0.215 **	(−0.119 **, 0.046)	0.007	−0.299 *	(−0.196 *, 0.089)	0.041						

* *p* ≤ 0.05, ** *p* ≤ 0.01, (r) correlation coefficient.

## Data Availability

Data are available upon request.
